# Subliminal Priming—State of the Art and Future Perspectives

**DOI:** 10.3390/bs8060054

**Published:** 2018-05-30

**Authors:** Mohamed Elgendi, Parmod Kumar, Skye Barbic, Newton Howard, Derek Abbott, Andrzej Cichocki

**Affiliations:** 1 Faculty of Medicine, University of British Columbia, Vancouver, BC V6T 1Z3, Canada; 2 BC Children’s & Women’s Hospital, Vancouver, BC V6H 3N1, Canada; 3 School of Electrical and Computer Engineering, University of British Columbia, Vancouver, BC V6T 1Z4, Canada; 4 College of Engineering, Madda Walabu University, Bale Robe 247, Ethiopia; kumaparm@isu.edu; 5 Department of Occupational Science and Occupational Therapy, University of British Columbia, Vancouver, BC V6T 2B5, Canada; skye.barbic@ubc.ca; 6 Nuffield Department of Surgical Sciences, University of Oxford, Oxford OX1 2JD, UK; newton.howard@nds.ox.ac.uk; 7 School of Electrical and Electronic Engineering, The University of Adelaide, Adelaide SA 5005, Australia; derek.abbott@adelaide.edu.au; 8 Centre for Biomedical Engineering, The University of Adelaide, Adelaide SA 5005, Australia; 9 Skoltech Center for Computational and Data-Intensive Science and Engineering, Skolkowo Institute of Science and Technology, Moscow 143026, Russia; cia@brain.riken.jp; 10 College of Computer Science, Hangzhou Dianzi University, Hangzhou 310000, China; 11 Department of Informatics, Nicolaus Copernicus University, 87-100 Torun, Poland

**Keywords:** subliminal priming, social psychology, persuasion, marketing, advertisement, event-related brain potentials, subliminal perception, affective priming

## Abstract

The influence of subliminal priming (behavior outside of awareness) in humans is an interesting phenomenon and its understanding is crucial as it can impact behavior, choices, and actions. Given this, research about the impact of priming continues to be an area of investigative interest, and this paper provides a technical overview of research design strengths and issues in subliminal priming research. Efficient experiments and protocols, as well as associated electroencephalographic and eye movement data analyses, are discussed in detail. We highlight the strengths and weaknesses of different priming experiments that have measured affective (emotional) and cognitive responses. Finally, very recent approaches and findings are described to summarize and emphasize state-of-the-art methods and potential future directions in research marketing and other commercial applications.

## 1. Introduction

**Figure 1:**
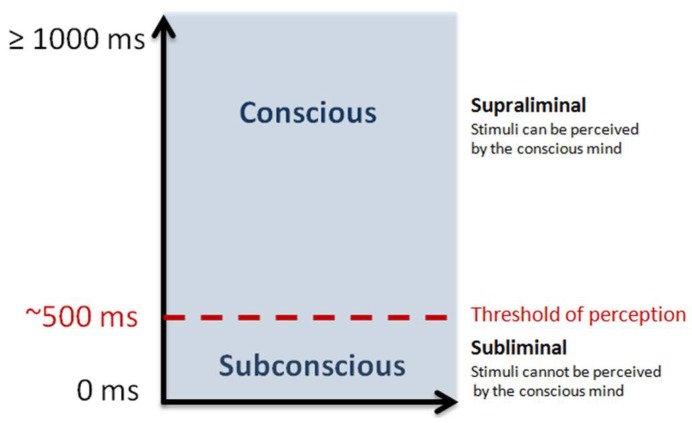
An illustration of supraliminal vs. subliminal priming. In subliminal priming, subjects are not aware of the stimuli as it occurs quickly (approximately less than 500 ms), yet it still influences them.

*Priming* refers to an increased sensitivity to certain stimuli, resulting from prior exposure to related visual or audio messages [1]. When an individual is exposed to the word “cancer”, for example, and then offered the choice to smoke a cigarette, we expect that there is a greater probability that they will choose *not* to smoke as a result of the earlier exposure. *Subliminal priming* occurs when an individual is exposed to stimuli below the threshold of perception [2], as detailed in Figure 1. This process occurs outside the realm of consciousness and is different from memory which relies on direct retrieval of information.

Subliminal priming is established based on a “primed” stimuli that is below the threshold of conscious detection [3]. Previous literature highlights that information below the threshold of conscious detection can elicit “diffuse processing” compared to information above the threshold [4]. Diffuse processing occurs when the stimuli spill over onto a temporally adjacent stimulus. For example, subliminally presented smiling and scowling faces have been shown to positively and negatively shift evaluative judgments of subsequently-presented affectively-neutral Chinese ideographs [5].

In the clinical and research contexts, subliminal priming depends on the specific indicators sampled and the time-frames over which they are measured [6]. Subliminality may vary over time as a function of dispositional factors and environmental variables. As a result, a strong conceptual and measurement model is needed to understand, study, and apply this concept.

**Figure 2:**
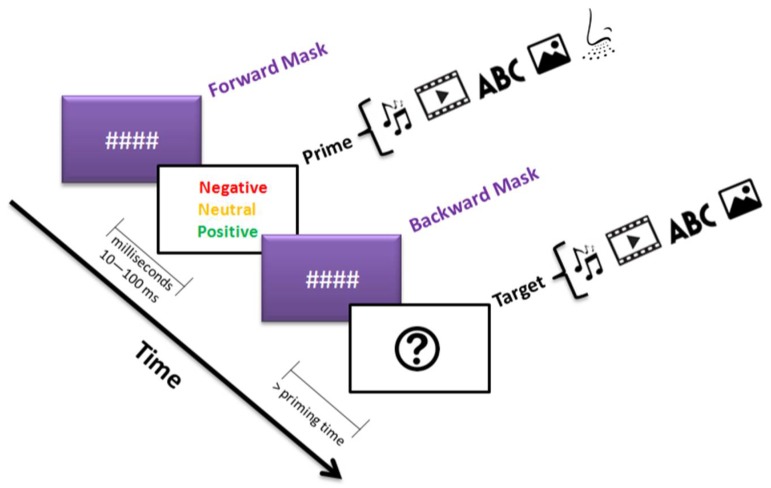
An example of a typical subliminal priming trial. The arrow depicts the flow of time. The priming process shows how exposure to one stimulus (e.g., audio, video, words, or images associated with a negative, neutral, or positive emotion) influences the response to the target, which is another stimulus. The symbols #### represent forward and backward masks. Masking is a widely used and powerful way of studying visual processes to reduce (or eliminate) any influence from previous or upcoming primes.

Subliminal priming has been studied extensively in psychological research, often for the purposes of market research. In cognitive psychology studies, subliminal priming study methodologies often include very short experimental observation periods (milliseconds) to understand the impact of a brief exposure on an individual’s decision-making when exposed to a subsequent stimulus (see Figure 2) [7]. The methods and results emerging from these studies are increasingly being used in the social and health sciences, including for advertising, human–computer interactions, and political campaigns. A thorough understanding of subliminal priming is essential to optimize the outcomes and performance of this research across the sciences. Providing an organized overview on the topic will be of use to researchers looking to understand the current body of research in the area.

**Figure 3:**
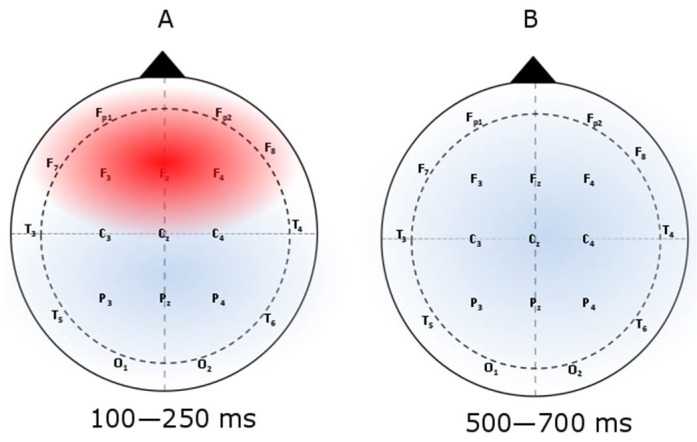
The impact of subliminal priming on ERP. The topographic maps [10] confirm the results of overall priming effects (i.e., ERPs with primed pictures minus ERPs with unprimed pictures): (**A**) 100–250 ms at the frontal lobe and (**B**) 500–700 ms at the parietal lobe. There is a significant difference between primed and unprimed ERPs within the 100–250 ms duration at the frontal lobe.

Subliminal priming is complex [8]. In our overview, we discuss conceptual models and contemporary studies of subliminal priming, with implications for empirical researchers as well as applied psychologists who use these findings in advertising, marketing, persuasion/attitude change, and other areas. There is an increased interest in understanding the depth and effectiveness of subliminal priming from psychological and physiological perspectives [9]. Understanding and predicting the psychological states of participants in real-time in response to subliminal priming should theoretically improve the impact of its effect. Physiological signals, such as electroencephalograms (EEGs) and eye-based measures (eye tracking), can be used to measure psychological responses. Current research on subliminal priming, including both psychological and physiological mechanisms, can be divided into the following five categories, which make up the sections of this review:

1. Types of priming
2. Effective priming
3. Design protocol for priming studies
4. Neurological impact of priming (examines prior studies that discuss the neurological basis of subliminal priming). This includes event-related potential (ERP) (e.g., P300), N1, and how priming effects these components, and the evaluation of various stimuli and their impacts on ERPs. The impacts of priming on ERPs of 100–250 ms are shown in Figure 3.
5. Eye tracking
6. Advertising

### 1.1. Types of Priming

#### 1.1.1. Semantic Priming

Semantic priming occurs when the prime and target words are semantically related and share features [11]; for example, the word “dog” semantically primes the word “wolf”, as both refer to similar animals.

#### 1.1.2. Visual Priming

Visual priming relies on visual stimulation without the use of other stimuli types, such as semantic or verbal [12,13,14]. *Masked priming* is a type of visual priming and results in only 14% of objects that are named. The term “masked” refers to the symbols “####” displayed before or after the prime. An example of masked priming is shown in Figure 2. When the same images are shown again about 15–20 min later without repetition, naming accuracy has been shown to increase by nearly 35%. Visual priming has been shown to last longer than semantic priming, in terms of its influence on the subject, and is resilient against stimuli intervening the prime and the intended target word [15].

Visual and semantic priming using ‘supraliminal’ objects has been found to last longer than those using subliminal objects alone [16]. Supraliminal priming is a type of priming that aims to influence behavior unconsciously. Supraliminal primes have been shown to have stronger and longer-lived effects on behavior, compared to subliminal primes [17].

After discussing visual priming, it is important to mention the *subliminal mere exposure* (SME) effects. SME is defined as the enhanced liking for stimuli following repeated subliminal exposures to those stimuli as a form of visual priming. In other words, preferences for an item (an object) can be formed after repeated exposure to the same subliminal prime [18]. Interestingly, the SME effect is actually significantly stronger than the mere exposure effect for stimuli that are consciously perceived [19]. The SME effect is particularly well-established and relevant for advertising and marketing [20].

#### 1.1.3. Response Priming 

Response priming is a unique type of subliminal priming where the exposure to the prime and stimuli occur in rapid succession [21]. Using rapid succession intervals (e.g., less than 100 milliseconds), the prime and target are presented and then paired with alternative or identical motor responses [22,23]. The subject’s motor response that classifies the target stimulus can be interfered when a prime is presented in conflict with the target [21]. For example, when a prime word (i.e., stimuli) is presented to the “left” of the subject but the text reads to the “right”, the degree to which priming affects the response is completely independent of the subject’s visual awareness of the prime [24,25].

The interval between the onset of the prime and the stimulus is called stimulus-onset asynchrony [26]. The measurable effects in response priming experiments are defined as the *response time* and *error rate* [26]. The response time is the time taken by the subject to react to the target stimuli [26], and it can be improved by exposure to consistent primes—exposure to inconsistent primes negatively affects the response time [26]. The error rate measures the impact of a prime on the subject’s recognition of the target [26].

#### 1.1.4. Perceptual and Conceptual Priming. 

Perceptual priming is concerned with physical correlations between the properties of the target stimuli and the prime. Biederman and Cooper [27] showed that the magnitude of perceptual priming is independent of the object’s size, but the reaction times and error rates for identical responses in old-new shape judgments were increased by changes in object size. Jolicoeur [28] stated that the “recognition” latency of a particular shape is lengthened with variation in its size. This, along with other studies [29,30,31,32,33,34] that have calculated time costs for recognition, have led to the notion that different shapes are stored in the memory on a specified scale [35,36]. The current design of perceptual priming experiments is based on earlier documentation [14], which suggested that the time required to recognize previously perceived objects decreases through subsequent exposure. Experiments conducted in one study [27] showed that the orientation/alignment of the object likely has no effect on object-naming performance.

Conceptual priming uses semantic tasks to enhance the meaning of a stimulus. For example, if the word “t-shirt” is presented, it will have priming effects on the word “shoes”, since both words fall into the same semantic category [37]. Based on the cited evidence, it can be argued that the difference between perceptual and conceptual priming lies in whether items with a similar form or meaning are primed.

#### 1.1.5. Positive and Negative Priming 

Positive and negative priming occurs when there is a change in the speed at which stimuli are processed. Positive priming accelerates processing and occurs when a stimulus is simply experienced, regardless of whether it is consciously perceived [38]. One study [39] showed differences between reactions to recently ignored stimuli and control stimuli; reactions to ignored stimuli were slower and more prone to error. This phenomenon was termed the *negative priming effect*, because it reduces the processing speed [39]. Bentin et al. studied the effects of positive and negative priming on ERPs [40].

Negative priming has been explained using four theoretical approaches. The two most commonly accepted models are (1) distractor inhibition and (2) episodic retrieval. The distractor inhibition model [41] posits that distractors in the form of previously ignored stimuli are actively inhibited and therefore, enable the selection of the target. When using a previous distractor as a new target, the response is likely to be hindered due to target inhibition.

An *episode* is defined as an encounter with a stimulus that is stored in the memory separate from other encounters. The episodic retrieval model states that each episode contains information about the stimulus as well as the given response. On this basis, one study [42] found that negative priming occurs when retrieving the prime episode while exposed to the probe stimulus.

#### 1.1.6. Associative and Context Priming.

Associative priming occurs when the prime and target are associated but not necessarily related through semantic features. For example, the word “dog” can be used as an associative prime for “cat”, because these words frequently appear together (in phrases such as “it is raining cats and dogs”) and are closely associated.

Context priming is said to occur when a word is used to accelerate the processing of stimuli that are likely to occur within the given context [43]. For example, a study showed that the assigned polling locations (e.g., a church or school) can influence how people vote [44].

#### 1.1.7. Olfactory Priming 

Olfactory Priming occurs when the odor prime is used to influence the evaluation of the target stimuli [45]. While other priming types are well investigated in the literature, few studies exist to aid in the understanding of olfactory priming. For example, a recent study showed unpleasant odour had aneffect on the ratings of faces presented simultaneously [46].

## 2. Subliminal Priming Factors

Two essential components for effective subliminal priming are (1) contamination and (2) emotional and neutral stimuli.

### 2.1. Minimization/Removal of Contamination

Several studies [47,48,49,50] have found that the effect of subliminal priming may be contaminated by a stimulus that is consciously perceived (i.e., the participant is aware of it). For example, one study found that stimulus exposure times of 33 ms were not short enough to ensure unconscious processing, as some participants in a separate study [50] were able to detect faces within this duration. Setting a duration threshold that ensures the maximum number of participants will not detect the subliminal stimuli will help reduce contamination. The threshold value can be determined by using current standards, or by conducting a smaller pilot study to predetermine the required threshold. For example, Potter et al. [51] found that people can detect meaning in rapid serial visual presentations at a threshold level of 13 ms.

### 2.2. Differences between Emotional and Neutral Stimuli

Emotion improves memory processing, regardless of consciousness, as seen in a study by Karremans et al. [52]. Another study [53,54] investigated how exposure to words that intentionally generate positive, negative, and neutral emotional responses can change behavioral and electrophysiological states. The results revealed that subjects preferred images linked to a positive affect condition (e.g., joy, happy, etc.), compared to those linked to a negative affect condition. Note that this difference was not significant for the behavioral response data. Using electrophysiological responses, Lang et al. [55] found that there is a difference between emotional and neutral facial expressions. Examples of words used for priming are as follows: positive words (juicy, cherry), negative words (fungus, bitter) and neutral words (jacket, curtain).

## 3. Design Protocol

In this section, examples are provided regarding the exposure/response times accompanying stimuli in each type of priming. Table 1 summarizes the design protocol in terms of priming type, exposure/response times, and stimuli types.

## 4. Electrophysiological Impact

**Figure 4:**
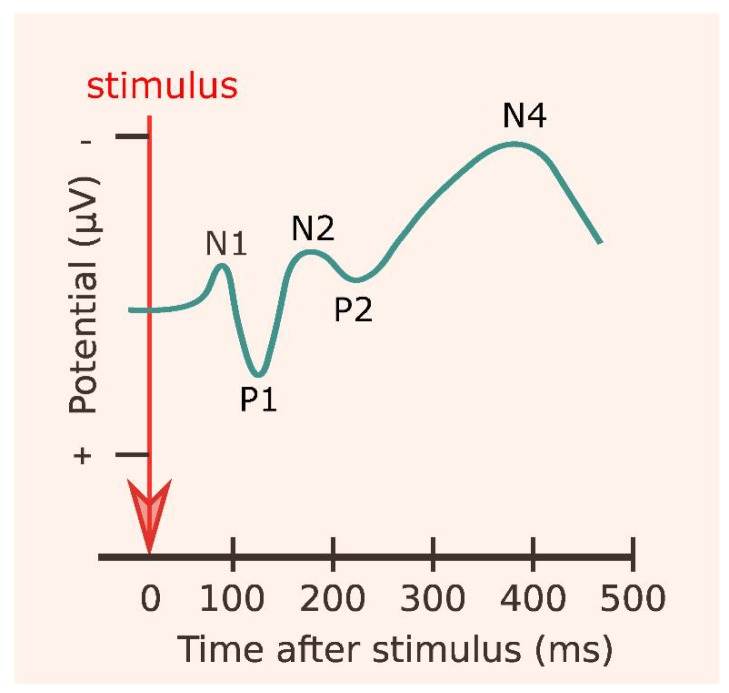
An example of event-related potentials. N400 or N4 wave refers to the negative deflection peaking around 400 ms after the stimulus. Note that the negative peak is above zero and the positive peak is below zero as averaging across multiple trials cancels out random variations [77].

Event-related potentials (ERPs) are brain signals that arise as the result of a thought, internal stimuli, or an individual’s perception of an external stimulus. ERP signals can be measured through EEG signals [75], and ERPs involve multiple neurological processes, such as memory, expectation, attention, and/or changes in mental state. Note that the crucial features that EEG signals are event related (i.e., time locked to stimulus onset) and averaged over many trials. ERPs consist of two components: (1) type of polarity, and (2) number of milliseconds after the onset of the stimulus. For example, a negative-going peak, which is the first measurable peak in the ERP waveform (occurring about 400 ms after the stimulus onset), is called N400 or N4 [76]. Note that the negative peak is above zero and the positive peak is below zero as averaging across multiple trials cancels out random variations [77], as can be seen in Figure 4.

One study [78] considered the effects of subliminal emotion words on the preferences and judgments towards subsequent visual target stimuli (i.e., paintings and portraits) [78]. The stimuli were distinguished via the following criteria:

1. Positive (arousing), such as “happiness” and “wonder”;
2. Relatively negative (arousing), such as “brutality” and “danger”;
3. Positive (non-arousing), such as “mild”;
4. Negative (non-arousing), such as “laziness” and “lethargy”.

The subliminal responses to those words were measured using ERPs. The ERPs were then used to investigate the timing and effects of priming on subjects. In multiple studies [79,80,81], ERPs were also used to evaluate the effects of positive and negative subliminal primes on perceptual vigilance and defense responses. Table 2 summarizes the ERP studies based on components, stimuli, and locations.

Other ERP studies [82,83,84] have examined affective processing using emotionally arousing pictures. Based on these studies, an index of around 300 ms following exposure to a stimulus was suggested, which is believed to facilitate the processing of emotional stimuli in the visual cortex. Another study [84] reported a late-range ERP correlate of emotional processing, which has been termed the late positive complex (LPC). The LPC effect arising from emotion starts at around 400 ms after the stimulus onset, and the effect has been held responsible for activating motivational systems in the brain via emotional stimuli [85]. The lateralized readiness potential (LRP) [86] monitors the development of hand-specific motor activation. LRPs have been used [87] to demonstrate that subliminal priming can successfully activate a partial response in a number-assignment test.

ERP research can build an understanding of the impact that participants’ stimuli processing has on their mental and physical conditions. Analyses of ERPs seem to suggest that the priming process can be divided into two categories as follows [88]: (1) early ERP waves, within the first 100 milliseconds after stimulus; and (2) tater ERP waves reflect the manner in which the subject evaluates the stimulus in terms of cognition.

Perhaps the question now is, can ERP capture the mood changes in a priming experiment, and if this is the case, which component in the ERP needs to be examined to quantify the mood change? A study [89] suggested that variations in mood during priming experiments can be examined after repeated exposure to positive or negative stimuli. However, other studies [90,91] have indicated that positive mood changes are associated with heuristic processing of stimuli (i.e., processing based on past experience and memory), whereas negative mood changes are associated with analytic processing. These studies concluded that prolonged exposure to positive or negative stimuli influences people’s affective states (e.g., motivation, arousal, and valence) and processing styles (e.g., auditory and visual). Unpleasant words were shown to elicit a higher amplitude (i.e., demonstrably stronger ERPs) in the ERP components P100 (P1), N100 (N1), P200 (P2), and P300 (P3) [92], suggesting that higher amplitudes may indicate negative priming (i.e., the presence of negative stimuli). ERPs have also been used to assess attitudes towards presented stimuli [93], providing further evidence that a direct correlation between the types of stimuli and the resulting ERPs exists.

In summary, varying methodologies (i.e., modality and valence of stimuli, experimental design, number and location of recording electrodes, and ERP components evaluated) have been used. However, the most consistent finding among the above-cited studies is that emotional stimuli can elicit the ERP component P3 [88]. Other studies [55,94,95] reported that the N1 and P2 components can be differentiated according to pleasant and unpleasant word categories, measured at electrode P3 in the EEG measurement system. These results can be used to detect whether the priming type is positive or negative.

Additionally, semantic priming effects were obtained from the N4 ERP component, which is considered an electrophysiological index for semantic priming [96]. Before exposure to the prime, a semantic task set was presented, which differed from the perceptual task set that had previously been shown to attenuate N4 priming. The results were comparable regardless of task difficulty and type (nonverbal vs. verbal). The following propositions can be made based on previous studies [97,98].

Automatic processes

- Act independently from capacity-limited attention resources
- Are not susceptible to interference from other processes
- Can perform in parallel to other processes
- Are unconscious

Although there is no direct empirical support available for the above propositions, this classical view is inherent in recent theories on automaticity, which strongly influences contemporary conceptions of cognitive control [99].

Automatic processes are not subject to the influences of attention, cognitive resources, or task demands; otherwise, they fall under the category of controlled processes. Several studies [100,101,102] have posited that if behavioral or neurophysiological effects associated with semantic processing are only obtained for an attended word, then the semantic processing is controlled. Evidence for controlled processing can be found in a subject’s attention to meaning rather than letter structure [103].

### 4.1. The N4 Priming Effect

The main characteristic of an N400 (or N4) event is its negative deflection that peaks at around 400 milliseconds (within the time window of 250 milliseconds to 500 millisecond) after the onset of the stimulus. Compared to unrelated pairings, the amplitude of the N4 component is less negative in combinations that are semantically related, which is referred to as the N4 priming effect [40,104,105]. Interestingly, both consciously and unconsciously perceived primes modulate the N4 amplitude [106,107]. In ERP studies, semantic priming effects are reflected by amplitude changes in the N4 ERP component [107].

To investigate how task sets modulate unconscious automatic processes, a novel experimental paradigm was developed by Kiefer and Martens (2010) [96]. In a previous study [108], this experimental paradigm was applied, and participants were asked to engage in a semantic or perceptual induction task before receiving masked semantic priming through a lexical decision task. The induction task was expected to activate a corresponding task set (semantic or perceptual) and modulate sensitivities to semantic and perceptual processing pathways. Within a few hundred milliseconds after the task was started, it was observed that a semantic induction task could enhance unconscious semantic priming, whereas a perceptual induction task should attenuate semantic priming. This divergence between behavioral and N4 measures has been observed in several other studies on semantic processing [107,109].

### 4.2. Motor Responses and the N4

The first action-sentence compatibility effect (ACE) cortical measurements of semantic processing and motor response have been discussed in a previous study [110]. The findings from this study suggest that there is a coupling between motor mechanisms and action-sentence comprehension. The presence of N400-like effects suggests an incompatibility with motor processes, which interferes with sentence comprehension.

The general components of motor responses, such as direction, have been investigated in past studies [111]. One such study [110] explored neural markers in the motor–language relationship and found an N400-like effect, which indicates that the motor process may interfere with semantic sentence comprehension.

Based on these studies, it can be concluded that sentence and motor information are integrated bidirectionally. ERPs offer a precise tool for measuring time (i.e., in milliseconds) and they include recordings of ongoing electrophysiological activity using electroencephalography (EEG). It is important to note that ERPs occur when various neural activities are activated in response to cognitive, sensory, and motor inputs [112]. The N4 component tracks semantic processing, and it has been found that at approximately 400 ms after the presentation of a word (stimulus), a large, negative deflection occurs in the ERP [88]. When integration of a stimulus into a previous semantic context is difficult, the N4 is typically larger [113]. The N4 priming effect has been reported with semantic violations in language and processing of other meaningful stimuli [108,114,115].

This section discussed the significance of ERPs in priming studies. ERP components, which can be used to identify priming types, were defined, as well as the positions of electrodes in different parts of the brain where these components are retrieved.

## 5. Eye Tracking and Subliminal Priming

During the late 19th century, French ophthalmologist Louis Émile Javal first described eye movement while reading [126]. Javal concluded that the eyes make rapid, short movements with brief pauses and do not continuously move along a line of text. Most notably, Javal made these observations using just his own vision. His discovery has since been confirmed in recent studies with current eye-tracking technology [127,128].

In 1967, Yarbus [129] found that tasks given to participants influence their eye movements, while recent research has proposed that conscious processing of stimuli does not influence initial eye movements [130,131,132]. There have been several studies on eye tracking and subliminal priming; for example, Balcetis and Dunning [133] confirmed that the rapid eye movements after exposure to visual stimuli can be used as an indicator for how a participant has interpreted a given stimulus.

Caspi et al. [134] investigated the impact of subliminal primes for death on eye movements. They proposed that the primes may serve as a “knob” that shifts one’s gaze towards, or away from, visual stimuli. Caspi and colleagues specifically showed that this was related to “terror management” defenses.

Fromberger et al. [135] investigated sexually preferred stimuli, finding that the fixation time was longer for preferred than non-preferred stimuli. When a sexually preferred stimulus was concurrently presented with a non-preferred stimulus, initial fixation was more often directed towards the preferred stimulus. For the first time, these results demonstrated an attention bias towards sexually preferred stimuli.

Other studies [136,137,138,139] validated parallelism in priming experiments. Parallelism is when components of a sentence are grammatically the same or similar in terms of their construction, meaning, or meter [140,141]. Parallelism can be demonstrated by having two sentences linked by a coordinating conjunction. In this case, the second clause is read faster when it is similar in syntactic structure to the first clause. This phenomenon is known as the *parallelism effect* [140,141]. Eye-tracking experiments were used to investigate (a) the duration of parallelism effects, (b) their presence in the absence of verb repetition, and (c) their potential modulation caused by the meanings of coordinating conjunctions.

Parallel language activation and the impacts of language proficiency and lexical status were examined by Blumenfeld and Marian [142]. In this study, bilingual German and English speakers were separated into two groups based on their first language. Translationally equivalent and non-equivalent target words were presented, and the participants were asked to identify targets (e.g., a hen) from displays that included similar-sounding German competitor words (e.g., “hemd”, meaning “shirt”). Native German speakers’ eye movements were used to index the co-activation of German words. The results showed that both groups of bilingual speakers co-activated German while processing translationally equivalent targets. However, only bilingual participants who spoke German as a first language co-activated German while processing English targets. These results suggest that high language proficiencies and translational equivalencies boost parallel language activation in bilinguals.

Another type of eye tracking study is where the influence of subliminal visual information on eye movement is investigated [143]. Van der Stigchel and colleagues studied how a subliminal distractor was placed in participants’ peripheral fields of vision while they made upward and downward eye movements. These effects were found to be minimal when compared to a control experiment that presented a supraliminal distractor. This is an area of study that requires further examination.

In regard to people who have had a stroke, eye-tracking and priming has been used to investigate lexical processing in Broca’s (left inferior frontal gyrus) and Wernicke’s (left posterior superior temporal gyrus) areas of the brain in people with aphasia (i.e., people who are unable to speak, but can understand others when they speak, usually due to a stroke). In a study, participants were studied by examining the duration of fixation on rhymes (e.g., “carrot-parrot”) and cohorts (e.g., “beaker-beetle”). When compared to an age-matched control group, Mirman et al. [144] found larger rhyme effects in Broca’s aphasic participants and larger cohort effects in Wernicke’s participants. Across all aphasic participants, a negative correlation was found between rhyme and cohort competition effects. Conventional explanations for these results assert that subjects with Broca’s or Wernicke’s aphasia have two different impairments, thus giving rise to varied patterns of rhyme and cohort competition. Contrary to this argument, an analysis of data unique to each participant with aphasia revealed that rhyme and cohort competition effects are negatively correlated. This suggests that these effects may not be completely independent.

Finally, in this section, we summarize other subliminal priming studies [122] that have measured horizontal and vertical eye movements/blinks using electrodes placed below and to the sides of the eyes, recorded simultaneously with EEG signals. Researchers have hypothesized the importance of measuring eye movement in subliminal priming experiments. It has been verified that eye tracking has been successfully applied in prior research to the study of a wide variety of phenomena related to attention and vision [145]. Therefore, eye tracking is crucial to current research and can provide extra information, besides ERPs, about the influence of subliminal priming, as discussed in Section 3 and Section 4.

## 6. Subliminal Priming and Advertisements

Several recent studies [146,147,148] have explored the effects of priming on consumer behavior and brand impression. The authors of the present article came across contrasting results. One study [52] assessed how priming for a beverage brand name affects an individual’s choice of beverage. The findings indicated that priming had a positive impact on the participants choice. On the other hand, other studies [149,150] have pointed out that there is no reliable evidence to support the more sensational claims regarding the power of subliminal influence on consumers. Under these contrasting views, the following subsections describe recent findings.

### 6.1. Studies Validating the Impact of Priming

The concept of “subliminal messages” was unknown until 1957, when marketers claimed its potential use for persuading consumers [151,152,153]. A study conducted by Karremans et al. [52] verified an earlier claim made by James Vicary, who used subliminal priming to boost coca-cola and popcorn sales in a movie theatre by flashing messages across the screen. The results of Vicary’s experiment revealed that there was an 18.1% increase in coca-cola sales and a 57.1% increase in popcorn sales. The study [154] explored two aspects of consumer responses to subliminal priming:

- Whether consumers’ choices were affected by priming
- Whether consumers’ choices were moderated by their individual feelings of thirst

The findings [154] included the following: (a) priming consumers with the name of a thirst-quenching beverage makes it more likely that they will choose that beverage; and (b) priming also increases their intention to choose the brand, but only for individuals who are thirsty. These studies [151,155,156,157] also cited comparable results from popular examples of priming in media. For example, one study [158] investigated the presence of hidden, Satanic messages in rock music. The study answered two questions: (a) whether these messages could be attributed to active construction on the part of the listener and (b) whether the content was present in the recordings. The results revealed that 75% of participants reported having heard the controversial messages, and 44% believed that record companies and recording groups hid these messages in recordings.

Karremans et al.’s study [52] further stated that subliminal priming should be directly related to a goal to be effective. For example, one consumer was primed to buy iced tea using the prime word “thirsty”, which positively affected the consumer’s choice to choose the brand. Karremans et al. [52] also added that subliminal messages can help consumers fulfill a goal, but only if they already had that goal; that is, a consumer will only buy a product if they already had the intention of doing so [159,160]. Findings by Dijksterhuis et al. [161] and Strahan et al. [158] suggest that priming with goal-relevant cognitions will lead to the enhanced effectiveness of an advertisement. It seems that priming not only affects choice, but also intention, which has been proven to be the best indicator for real-world behavior.

### 6.2. Studies Negating the Impact of Priming

Market research [162] shows that in the United States alone, consumers spend more than 50 million dollars annually on self-help audiotapes (SHAs) containing subliminal messages. The industry is comprised of products that improve memory, boost self-esteem, and facilitate weight loss. However, previous studies have failed to establish evidence for these subliminal messages [163,164,165,166].

In studies conducted on subliminal SHAs, tapes for improving memory and self-esteem were selected. There were two reasons for this choice: (1) participants were easily available for these experiments and (2) several well-established measures were available for the pre-test and post-test designs. The predicted results were higher post-test scores for memory and self-esteem; however, the actual results were the exact opposite for both categories. Other studies [167,168,169,170] found no actual change to perceived effects on self-esteem and memory, yet many participants who volunteered with the hope of improving these abilities ended up believing that the tapes had been effective. The impact of SHAs on perceived improvement was determined to be an illusory placebo effect [168,170].

Although other scientific studies [171,172,173,174,175] have pointed out therapeutic and self-improvement effects, none have been published in peer-reviewed journals. Also, many of these studies were conducted by SHA manufacturers, which casts doubt over the authenticity of the results.

### 6.3. Popular Cases of Subliminal Priming in Media and Advertisement

Below are some popular cases in which the concept of subliminal priming has been used within different contexts and applied situations:

- **Sports**: Ferrari’s F1 cars displayed a barcode that was criticized for subliminally flashing the logo of its sponsor company, Marlboro [176]. The barcode was also in violation of the ban on tobacco advertising, and therefore, Ferrari removed the design in 2010 [177].
- **Politics**: During the 2000 US presidential campaign, former President George W. Bush used priming to gain an advantage over his Democratic rival, Al Gore [178]. The Bush campaign launched a television ad containing a frame with the word “RATS” in a scaled-up font size. The Federal Communications Commission (FCC) investigated this matter, but no penalties were issued in this case.
- **War**: Priming was applied to a scientific instrument called the tachistoscope, which projected images over an extremely brief period. The tachistoscope was used to improve soldiers’ reading speeds and test their eyesight during World War II.
- **Music**: British band Judas Priest was prosecuted for including subliminal messages in their songs, which resulted in suicide attempts by two young men [179]. The songs alleged to have an influence on their behavior were “Do It” and “Better By You, Better Than Me”. The judge was not convinced that the hidden messages existed, and the band was acquitted. The judge decided that the young men could not have attempted suicide unless they had the real intention of doing so, or that the messages could not have been perceived without the “power of suggestion”.
- **Movie and TV shows:** Product placement in films and television shows represents a form of subliminal/incidental advertising. A prime example of placing a product in a movie to subliminally increase sales was the placement of Ray Ban sunglasses in the Tom Cruise movie *Top Gun*. The brand became extremely popular when Tom wore them in the film [180].

There are many other examples of subliminal priming using the senses, including the use of odors for marketing and commercial purposes. The scent component is obviously important for hygiene, beauty and food products, but it can also affect other varied products. At the point of purchase, scent diffusers are commonly used to favor the quality of the customer experience, to reinforce the image of a brand by the diffusion of an odor related to its universe or to a particular product [181]. The application for olfactory priming in the fields of advertisement and marketing is promising and increasingly common. However, the evidence base for olfactory priming is limited. Future research is needed to understand the associated psychological and physiological processes responsible for the potential effects.

In summary, the future direction of subliminal priming has significant potential across the social and health sciences. Subliminal priming is effective and can influence decision-making. This review has summarized that it is critical to consider the design of the experiment when studying subliminal priming, as the design appears to play a major role in achieving more reliable findings. As shown in this review, subliminal priming can be used for investigating persuasion, human–computer interactions, elucidation of emotions, political campaigns, rehabilitation, 3D virtual tutoring systems, virtual intelligent tutoring systems and lie detection. The psychological and physiological mechanisms underpinning subliminal priming for advancing learning, health, marketing, and political outcomes are ongoing areas for study.

## 7. Conclusions

The main objective behind this review was to provide a summary of the latest developments in subliminal priming. This review discussed how priming is one of the most widely used concepts in product promotion and marketing across nearly all areas of public interest and described how subliminal priming with visual stimuli can be much stronger than subliminal priming with other sensory modalities. This review also stated that most scientific studies on priming are focused on the analysis of ERPs. ERPs can also serve as an objective method to assess the effectiveness of priming, particularly that of subliminal priming. ERPs consistently show high reliability in the evaluation, diagnosis, and categorization of priming responses found in different areas of the brain. This review also highlighted the critical importance of experimental design for ensuring precision and reliability. It is important for researchers to know how to set up an experiment in terms of exposure duration and stimuli. Areas of future study were also identified, including standardizing protocols and measurement/outcome approaches, more longitudinal subliminal priming studies across different populations and cultures, and focused study on unique populations such as children and people in developing countries. Together, this review provides a thorough summary of subliminal priming research and the potential for impact across a wide area of sciences, public health, marketing, and politics. We conclude that subliminal priming is a growing area of study that requires systematic and collaborative efforts to maximize its potential and impact.

## Figures and Tables

**Table 1:** Priming design parameters in different contexts.

TYPE OF PRIMING	EXPOSURE/ RESPONSE TIMES	STIMULI TYPES	REF.
**Positive and Negative Priming** ***A positive prime accelerates priming processing, while a negative prime slows priming processing.***	In word-naming experiments, participants began each trial by pressing the space bar on a keyboard placed in front of them. A blank interval of 250 ms followed the disappearance of the default display. The prime display then appeared and remained on the screen until the onset of the participant’s naming response. The prime display contained a single subliminal prime with exposure times of 33–200 ms. Longer prime exposure times allowed participants to identity the prime.	An overwhelming amount of priming research has used visual stimuli, but audio priming using human voices and artificial sounds has also been reported. Twelve high-frequency nouns served as target and distractor words on the prime and probe displays. The following words were used in several experiments: “BOARD”, “FLUTE”, “TABLE”, “PILOT”, “CLOUD”, “QUEEN”, “TIGER”, “GUEST”, “GLASS”, “PRIZE”, “BAKER”, and “CLERK”.	[38,39,40,41,42]
**Associative and Context Priming** ***(e.g., “sun” is an associative prime for “moon”.)***	The trials began by fixating a participant’s attention to the default display. In one study [56], the phrase “get ready” appeared in the center of the screen for 500 ms. After fixation, a 500-ms blank interval was presented, and then the prime appeared for 500 ms. Another 500-ms blank interval followed the prime, and then the target image appeared.	Primes can be the names of the pictures, the pictures themselves, or the names of the categories to which a picture might belong. Standard categories were provided in a previous study [57]. The targets can be pictures or black-and-white line drawings. One study [58] contained a standardized set of 260 black-and-white line drawings.	[43,56,57,58]
**Semantic Priming** ***(E.g., “Earth” is a semantic prime***	Three prime durations were tested (i.e., 100 ms, 250 ms, and 500 ms). The prime was immediately followed by the onset of a stimulus at the same location on the screen as that of the prime. In the experiments conducted in one study [59], the exposure times for semantically related primes ranged from 250 to 750 ms.	Both the target and prime are word stimuli, including two types of primes: Semantic but non-associative (e.g., dolphin and whale), Associative but non-semantic (e.g., spider web).	[60,61]
**Visual Priming** ***This is priming using visual cues.***	The exposure times for primes ranged from 42 to 56 ms (with an average of 47 ms), individually adjusted to each object based on pilot experiments.	The objects can be simple line drawings of tools, furniture, animals, clothes, vehicles, and other items, typically drawn with black, 2-pixel-wide lines on a white background.	[12,13,14]
**Response Priming** ***The prime and target are presented in quick succession, typically less than 100 ms apart.***	The intervals of prime exposure and stimulus-onset asynchrony (SOA) lasted 17‒100 ms.	Simple objects, like left and right arrows, can serve as prime and mask stimuli. Other examples include common shapes, such as squares, rectangles, hearts, and diamonds.	[22,23,24,25,62,63,64,65,66,67,68,69,70,71,72]
**Perceptual and Conceptual Priming** ***Perceptual priming is based on the form or format of the stimulus, while conceptual priming is based on the meaning of the stimulus.***	Perceptual: The subjects pressed a mouse button to start each trial. A fixation dot was presented for 500 ms, followed by either a 50- or 100-ms display of the object picture. The picture was followed by a 500-ms mask: a randomly appearing arrangement of straight and curved lines. Conceptual: Trials began with the 500-ms display of a fixation cross, followed by the target word for 2 s. Participants were instructed to read the word on the screen aloud. Two variations were used in the trials: Word association: Each trial began with the display of a cue word, and participants were asked to respond with the first word that came to mind. Category example: Participants were presented with a category name and asked to respond with the first member of that category that came to mind.	Perceptual: Simple line drawings of common objects may be used (e.g., plants or animals with basic names). The stimulus should consist of at least one pair with similar names but different shapes (e.g., a grand piano and an upright piano). Conceptual: For word association, standard word pairs can be used. The first word of each pair is designated as the cue, and the second word is the target (e.g., in “TUSK-elephant”, “TUSK” is the cue word and “elephant” is the target.	[73,74]
**Olfactory Priming** ***The odor prime influences the evaluation of the target***	A 3-s odor pulse was released, during which participants viewed a black screen.	Visual stimuli: neutral faces were used in the experiment. Out of the 18 female faces, nine were white/Caucasian, five were East Asian, and four were Afro-Caribbean. Out of the 18 male faces, 12 were white/Caucasian, five were afro-Caribbean and one was East Asian.	[45]

**Table 2:** Comparisons between event-related potential (ERP) studies based on components, stimuli, and locations. All ERP components consist of two components: type of polarity, and number of milliseconds after the onset of the stimulus. For example, a positive-going peak, which is the first measurable peak in the ERP waveform (occurring about 100 ms after the stimulus onset), is called P100 or P1.

Component	Stimulus	Summary	Location	Ref.
**P1, N1, P2, P3**	Mood adjectives (32 stimuli over 6 presentations). Subjects = 17. Duration: subliminal = 1 ms, supraliminal = 40 ms.	The study demonstrated that ERPs are sensitive to affective valences, whether consciously or unconsciously.	F3, F4, P3, P4, Cz, Pz, Oz	[92]
**P1**	Images of happy or fearful and surprised facial expressions (n = 140). Subjects = 17. Duration: primes (fearful or happy) = 30 ms, targets (surprised) = 800 ms.	Larger occipital P1 components were found with fearful rather than happy expressions.	A source analysis implicated the bilateral extrastriate cortex.	[116]
**N1, P2**	Threatening and neutral words were used as primes for groups with high and low social anxiety. The targets were images of neutral and angry facial expressions (n = 16). Subjects = 24. Duration: primes = 200 ms, targets = 500 ms.	The high social anxiety group showed attention bias after viewing the neutral primes but not the threatening primes, indicating suppression of the attention bias. The low social anxiety group demonstrated opposite effects.	O1/O2	[117]
**N2, P3**	Go/no-go task with subliminal primes (14 blocks of 72 arrow shapes; trials = 1008). Subjects = 21. Duration: primes = 16 ms, targets = 100 ms.	Inhibition-related ERPs were modulated as a function of prime congruency. The inhibition of impending motor responses can be initiated by unconscious stimuli.	The primes influenced frontal inhibitory control mechanisms.	[118]
**P2, P3**	Earthquake-(un)related words (12 of each category). Subjects = 24. Duration: primes = 17 ms, targets = 1500 ms.	More positive ERP deflections in related words than unrelated words.	P2: Posterior cingulate cortex; P300: parahippocampal gyrus	[118,119]
**P3**	Names of acquaintances in a lie detection protocol (5 known names, 4 unknown names). Subjects = 14. Duration: primes = 17 ms, targets = 150 ms.	Subliminal primes modulate ERPs when the task involves lying.	Fz, Cz, Pz, Oz	[120]
**P3**	Logic-based learning paradigms (3 magic squares of odd order). Subjects = 46. Duration: primes = 33.33 ms, targets = unknown.	Subliminal “cues” increased learner performances.	Not stated	[121]
**N1.9, P1.9, N4**	Repeated images preceded by masked image primes (n = 80). Subjects = 16. Duration: primes = 50 ms, targets = 300 ms.	Reduced amplitudes in N/P190 that may reflect early processing of object-specific representations. Changes in N400 reflect more domain-general sematic processing.	Posterior/anterior (locations not specified)	[122]
**N2.5, N4**	Animal names in streams of words with masked primes (numbers unknown). Subjects = 24. Duration: primes = 40 ms, targets = 300 ms.	N250 reflects processing at the level of forms, while N400 reflects processing at the level of meaning.	Parietal (CP1)	[123]
**P2, N4**	Concrete and abstract emotional words (720 German nouns). Subjects = 30.	Concreteness affected N400 and LPC.	FC3, FC4	[124]
**N4**	Action sentences (156 Spanish sentences: 104 related to hand actions, 52 neutral sentences).	N400 distinguished between compatible and incompatible primes and was more negative for incompatible primes.	Cz	[125]
**P6, N9**	Visual stimuli (3 ethnicities: white/Caucasian, East-Asian, and Afro-Caribbean) and olfactory stimuli (3 odor conditions: pleasant, unpleasant and a neutral control). Subjects = 20. Duration: primes = 3 s, targets = 300 ms.	Significant effects of odor were observed at 600 and 900 ms after face-onset	Left and right lateral frontal-temporal electrodes	[45]

## References

[1] Barutchu A., Spence C., Humphreys G.W. (2018). Multisensory enhancement elicited by unconscious visual stimuli. Exp. Brain Res..

[2] Kawakami N., Miura E., Nagai M. (2018). When you become a superman: Subliminal exposure to death-related stimuli enhances men’s physical force. Front. Psychol..

[3] Lourenco S.F., Ayzenberg V., Lyu J. (2016). A general magnitude system in human adults: Evidence from a subliminal priming paradigm. Cortex.

[4] Dehaene S., Cohen L. (2011). The unique role of the visual word form area in reading. Trends Cogn. Sci..

[5] Murphy S.T., Zajonc R.B. (1993). Affect, cognition, and awareness: Affective priming with optimal and suboptimal stimulus exposures. J. Personal. Soc. Psychol..

[6] Erdelyi M.H. (2004). Subliminal perception and its cognates: Theory, indeterminacy, and time. Conscious. Cogn..

[7] Greenwald A.G., Draine S.C., Abrams R.L. (1996). Three cognitive markers of unconscious semantic activation. Science.

[8] Saari T., Ravaja N., Laarni J., Turpeinen M., Kallinen K. (2004). Psychologically targeted persuasive advertising and product information in e-commerce. Proceedings of the 6th International Conference on Electronic Commerce.

[9] Ortells J.J., Kiefer M., Castillo A., Megías M., Morillas A. (2016). The semantic origin of unconscious priming: Behavioral and event-related potential evidence during category congruency priming from strongly and weakly related masked words. Cognition.

[10] Li B., Gao C., Wang W., Guo C. (2015). Processing fluency hinders subsequent recollection: An electrophysiological study. Front. Psychol..

[11] Ferrand L., New B. (2003). Semantic and associative priming in the mental lexicon. Mental Lexicon: Some Words to Talk about Words.

[12] Bar M., Biederman I. (1998). Subliminal visual priming. Psychol. Sci..

[13] Bartram D. (1974). The role of visual and semantic codes in object naming. Cogn. Psychol..

[14] Biederman I., Cooper E.E. (1991). Evidence for complete translational and reflectional invariance in visual object priming. Perception.

[15] Becker S., Moscovitch M., Behrmann M., Joordens S. (1997). Long-term semantic priming: A computational account and empirical evidence. J. Exp. Psychol. Learn. Mem. Cogn..

[16] Cave C.B. (1997). Very long-lasting priming in picture naming. Psychol. Sci..

[17] Francken J., van Gaal S., de Lange F. (2011). Immediate and long-term priming effects are independent of prime awareness. Conscious. Cogn..

[18] Yoshimoto S., Imai H., Kashino M., Takeuchi T. (2014). Pupil response and the subliminal mere exposure effect. PLoS ONE.

[19] Kwan L.Y.Y., Yap S., Chiu C.-Y. (2015). Mere exposure affects perceived descriptive norms: Implications for personal preferences and trust. Organ. Behav. Hum. Decis. Process..

[20] Stafford T., Grimes A. (2012). Memory enhances the mere exposure effect. Psychol. Mark..

[21] Kopp B., Mattler U., Goertz R., Rist F. (1996). N2, p3 and the lateralized readiness potential in a nogo task involving selective response priming. Electroencephalogr. Clin. Neurophysiol..

[22] Klotz W., Wolff P. (1995). The effect of a masked stimulus on the response to the masking stimulus. Psychol. Res..

[23] Klotz W., Neumann O. (1999). Motor activation without conscious discrimination in metacontrast masking. J. Exp. Psychol. Hum. Percept. Perform..

[24] Vorberg D., Mattler U., Heinecke A., Schmidt T., Schwarzbach J. (2003). Different time courses for visual perception and action priming. Proc. Natl. Acad. Sci. USA.

[25] Schmidt T., Vorberg D. (2006). Criteria for unconscious cognition: Three types of dissociation. Percept. Psychophys..

[26] De Groot A.M. (1984). Primed lexical decision: Combined effects of the proportion of related prime-target pairs and the stimulus-onset asynchrony of prime and target. Q. J. Exp. Psychol..

[27] Beiderman I., Cooper E.E. (1992). Size invariance in visual object priming. J. Exp. Psychol. Hum. Percept. Perform..

[28] Jolicoeur P. (1987). A size-congruency effect in memory for visual shape. Mem. Cogn..

[29] Besner D. (1983). Visual pattern recognition: Size preprocessing reexamined. Q. J. Exp. Psychol..

[30] Bundesen C., Larsen A. (1975). Visual transformation of size. J. Exp. Psychol. Hum. Percept. Perform..

[31] Ellis R., Allport D.A., Humphreys G.W., Collis J. (1989). Varieties of object constancy. Q. J. Exp. Psychol..

[32] Jolicoeur P., Besner D. (1987). Additivity and interaction between size ratio and response category in the comparison of size-discrepant shapes. J. Exp. Psychol. Hum. Percept. Perform..

[33] Larsen A. (1985). Pattern matching: Effects of size ratio, angular difference in orientation, and familiarity. Percept. Psychophys..

[34] Larsen A., Bundesen C. (1978). Size scaling in human pattern recognition. J. Exp. Psychol. Hum. Percept. Perform..

[35] Kosslyn S.M. (1987). Aspects of a cognitive neuroscience of mental imagery. Science.

[36] Ullman S. (1989). Aligning pictorial descriptions: An approach to object recognition. Cognition.

[37] Vaidya C.J., Gabrieli J.D., Monti L.A., Tinklenberg J.R., Yesavage J.A. (1999). Dissociation between two forms of conceptual priming in alzheimer’s disease. Neuropsychology.

[38] Reisberg D. (2007). Cognition: Exploring the Science of the Mind.

[39] Mayr S.B., Axel B. (2007). Negative priming as a memory phenomenon: A review of 20 years of negative priming research. J. Psychol..

[40] Bentin S., McCarthy G., Wood C.C. (1985). Event-related potentials, lexical decision, and semantic priming. Electroencephalogr. Clin. Neurophysiol..

[41] Tipper S., Driver J. (1988). Negative priming between pictures and words in a selective attention task: Evidence for semantic processing of ignored stimuli. Mem. Cogn..

[42] Neill W.T.V., Leslie A., Terry K.M., Gorfein D.S. (1992). Persistence of negative priming: Ii. Evidence for episodic trace retrieval. J. Exp. Psychol. Learn. Mem. Cogn..

[43] Stanovich K.E.W., Richard F. (1983). On priming by a sentence context. J. Exp. Psychol. Gen..

[44] Berger J., Meredith M., Wheeler S.C. (2008). Contextual priming: Where people vote affects how they vote. Proc. Natl. Acad. Sci. USA.

[45] Cook S., Fallon N., Wright H., Thomas A., Giesbrecht T., Field M., Stancak A. (2015). Pleasant and unpleasant odors influence hedonic evaluations of human faces: An event-related potential study. Front. Hum. Neurosci..

[46] Cook S., Kokmotou K., Soto V., Wright H., Fallon N., Thomas A., Giesbrecht T., Field M., Stancak A. (2018). Simultaneous odour-face presentation strengthens hedonic evaluations and event-related potential responses influenced by unpleasant odour. Neurosci. Lett..

[47] Williams M.A., Morris A.P., McGlone F., Abbott D.F., Mattingley J.B. (2004). Amygdala responses to fearful and happy facial expressions under conditions of binocular suppression. J. Neurosci..

[48] Fazio R.H., Olson M.A. (2003). Implicit measures in social cognition research: Their meaning and use. Annu. Rev. Psychol..

[49] Milders M., Sahraie A., Logan S. (2007). Minimum presentation time for masked facial expression discrimination. Cogn. Emot..

[50] Pessoa L., Japee S., Ungerleider L.G. (2005). Visual awareness and the detection of fearful faces. Emotion.

[51] Potter M.C., Wyble B., Hagmann C.E., McCourt E.S. (2014). Detecting meaning in rsvp at 13 ms per picture. Atten. Percept. Psychophys..

[52] Karremans J., Stroebe W., Claus J. (2006). Beyond vicary’s fantasies: The impact of subliminal priming and brand choice. J. Exp. Soc. Psychol..

[53] Mohan D.M., Kumar P., Mahmood F., Wong K.F., Agrawal A., Elgendi M., Shukla R., Ang N., Ching A., Dauwels J. (2016). Effect of subliminal lexical priming on the subjective perception of images: A machine learning approach. PLoS ONE.

[54] Kumar P., Mahmood F., Mohan D.M., Wong K., Agrawal A., Elgendi M., Shukla R., Dauwels J., Chan A.H. On the Effect of Subliminal Priming on Subjective Perception of Images: A Machine Learning Approach. Proceedings of the 2014 36th Annual International Conference of the IEEE Engineering in Medicine and Biology Society (EMBC).

[55] Lang S.F., Nelson C.A., Collins P.F. (1990). Event-related potentials to emotional and neutral stimuli. J. Clin. Exp. Neuropsychol..

[56] Matsukawa J., Snodgrass J.G., Doniger G.M. (2005). Conceptual versus perceptual priming in incomplete picture identification. J. Psycholinguist. Res..

[57] Battig W.F.M., William E. (1969). Category norms of verbal items in 56 categories a replication and extension of the connecticut category norms. J. Exp. Psychol..

[58] Snodgrass J.G., Vanderwart M. (1980). A standardized set of 260 pictures: Norms for name agreement, image agreement, familiarity, and visual complexity. J. Exp. Psychol. Hum. Learn. Mem..

[59] Shelton J.R., Martin R.C. (1992). How semantic is automatic semantic priming?. J. Exp. Psychol. Learn. Mem. Cogn..

[60] Perea M., Rosa E. (2002). The effects of associative and semantic priming in the lexical decision task. Psychol. Res..

[61] Neely J.H. (2012). Semantic priming effects in visual word recognition: A selective review of current findings and theories. Basic Processes in Reading.

[62] Ansorge U., Klotz W. (1998). Manual and verbal responses to completely masked (unreportable) stimuli: Exploring some conditions for the metacontrast dissociation. Perception.

[63] Ansorge U., Neumann O., Becker S.I., Kälberer H., Kruse H. (2007). Sensorimotor supremacy: Investigating conscious and unconscious vision by masked priming. Adv. Cogn. Psychol..

[64] Schmidt T. (2002). The finger in flight: Real-time motor control by visually masked color stimuli. Psychol. Sci..

[65] Schmidt T., Niehaus S., Nagel A. (2006). Primes and targets in rapid chases: Tracing sequential waves of motor activation. Behav. Neurosci..

[66] Schmidt T., Schmidt F. (2009). Processing of natural images is feedforward: A simple behavioral test. Atten. Percept. Psychophys..

[67] Mattler U. (2005). Flanker effects on motor output and the late-level response activation hypothesis. Q. J. Exp. Psychol..

[68] Leuthold H., Kopp B. (1998). Mechanisms of priming by masked stimuli: Inferences from event-related brain potentials. Psychol. Sci..

[69] Eimer M., Schlaghecken F. (1998). Effects of masked stimuli on motor activation: Behavioral and electrophysiological evidence. J. Exp. Psychol. Hum. Percept. Perform..

[70] Eimer M., Schlaghecken F. (2003). Response facilitation and inhibition in subliminal priming. Biol. Psychol..

[71] Mattler U. (2003). Delayed flanker effects on lateralized readiness potentials. Exp. Brain Res..

[72] Vath N., Schmidt T. (2007). Tracing sequential waves of rapid visuomotor activation in lateralized readiness potentials. Neuroscience.

[73] Postman L., Keppel G. (2014). Norms of Word Association.

[74] Voss J.L., Schendan H.E., Paller K.A. (2010). Finding meaning in novel geometric shapes influences electrophysiological correlates of repetition and dissociates perceptual and conceptual priming. NeuroImage.

[75] Brown C.M., Hagoort P., Brown C.M., Hagoort P. (1999). The Cognitive Neuroscience of Language.

[76] Castle P.C., Van Toller S., Milligan G.J. (2000). The effect of odour priming on cortical eeg and visual erp responses. Int. J. Psychophysiol..

[77] Watson N.V., Breedlove S.M. (2012). The Mind’s Machine: Foundations of Brain and Behavior.

[78] Gibbons H. (2009). Evaluative priming from subliminal emotional words: Insights from event-related potentials and individual differences related to anxiety. Conscious. Cogn..

[79] Bruner J.S., Postman L. (1947). Emotional selectivity in perception and reaction. J. Personal..

[80] Erdelyi M.H. (1974). A new look at the new look: Perceptual defense and vigilance. Psychol. Rev..

[81] Junghöfer M., Bradley M.M., Elbert T.R., Lang P.J. (2001). Fleeting images: A new look at early emotion discrimination. Psychophysiology.

[82] Schupp H.T., Cuthbert B.N., Bradley M.M., Cacioppo J.T., Ito T., Lang P.J. (2000). Affective picture processing; the late positive potential is modulated by motivational relevance. Psychophysiology.

[83] Schupp H.T., Junghöfer M., Weike A.I., Hamm A.O. (2003). Emotional facilitation of sensory processing in the visual cortex. Psychol. Sci..

[84] Schupp H.T., Stockburger J., Schmälzle R., Bublatzky F., Weike A.I., Hamm A.O. (2008). Visual noise effects on emotion perception: Brain potentials and stimulus identification. Neuroreport.

[85] Cuthbert B.N., Schupp H.T., Bradley M.M., Birbaumer N., Lang P.J. (2000). Brain potentials in affective picture processing: Covariation with autonomic arousal and affective report. Biol. Psychol..

[86] Gratton G., Coles M.G.H., Sirevaag E.J., Eriksen C.W., Donchin E. (1988). Pre- and poststimulus activation of response channels: A psychophysiological analysis. J. Exp. Psychol. Hum. Percept. Perform..

[87] Dehaene S., Naccache L., LeClec’H G., Koechlin E., Müller M., Dehaene-Lambertz G., van de Moortele P.F., Le Bihan D. (1998). Imaging unconscious semantic priming. Nature.

[88] Sur S., Sinha V.K. (2009). Event-related potential: An overview. Ind. Psychiatry J..

[89] Chartrand T.L., van Baaren R.B., Bargh J.A. (2006). Linking automatic evaluation to mood and information processing style: Consequences for experienced affect, impression formation, and stereotyping. J. Exp. Psychol. Gen..

[90] Bodenhausen G.V.K., Geoffrey P., Süsser K. (1994). Happiness and stereotypic thinking in social judgment. J. Personal. Soc. Psychol..

[91] Park J.B., Mahzarin R. (2000). Mood and heuristics: The influence of happy and sad states on sensitivity and bias in stereotyping. J. Personal. Soc. Psychol..

[92] Bernat E., Bunce S., Shevrin H. (2001). Event-related brain potentials differentiate positive and negative mood adjectives during both supraliminal and subliminal visual processing. Int. J. Psychophysiol..

[93] Crites S.L., Cacioppo J.T., Gardner W.L., Bernston G.G. (1995). Bioelectrical echoes from evaluative categorization: II. A late positive brain potential that varies as a function of attitude registration rather than attitude report. J. Personal. Soc. Psychol.

[94] Yee C.M., Miller G.A. (1987). Affective valence and information processing. Electroencephalogr. Clin. Neurophysiol..

[95] Roschmann R., Wittling W. (1992). Topographic brain mapping of emotion-related hemisphere asymmetries. J. Neurosci..

[96] Kiefer M., Martens U. (2010). Attentional sensitization of unconscious cognition: Task sets modulate subsequent masked semantic priming. J. Exp. Psychol.-Gen..

[97] Posner M.I., Snyder C.R.R., Solso R.L. (1975). Attention and Cognitive Control.

[98] Schneider W., Shiffrin R.M. (1977). Controlled and automatic human information processing: 1. Detection, search, and attention. Psychol. Rev..

[99] Van Elk M., van Schie H.T., Bekkering H. (2009). Short-term action intentions overrule long-term semantic knowledge. Cognition.

[100] Kellenbach M.L., Michie P.T. (1996). Modulation of event-related potentials by semantic priming: Effects of color-cued selective attention. J. Cogn. Neurosci..

[101] McCarthy G., Nobre A.C. (1993). Modulation of semantic processing by spatial selective attention. Electroencephalogr. Clin. Neurophysiol..

[102] Rees G., Russell C., Frith C.D., Driver J. (1999). Inattentional blindness versus inattentional amnesia for fixated but ignored words. Science.

[103] Henik A., Friedrich F.J., Tzelgov J., Tramer S. (1994). Capacity demands of automatic processes in semantic priming. Mem. Cogn..

[104] Kiefer M., Martens U., Weisbrod M., Hermle L., Spitzer M. (2009). Increased unconscious semantic activation in schizophrenia patients with formal thought disorder. Schizophr. Res..

[105] Kiefer M. (2005). Repetition priming modulates category-related effects on event-related potentials: Further evidence for multiple cortical semantic systems. J. Cogn. Neurosci..

[106] Deacon D., Hewitt S., Yang C.-M., Nagata M. (2000). Event-related potential indices of semantic priming using masked and unmasked words: Evidence that the n400 does not reflect a post-lexical process. Cogn. Brain Res..

[107] Kiefer M., Brendel D. (2006). Attentional modulation of unconscious “automatic” processes: Evidence from event-related potentials in a masked priming paradigm. J. Cogn. Neurosci..

[108] Ibáñez A., Manes F., Escobar J., Trujillo N., Andreucci P., Hurtado E. (2010). Gesture influences the processing of figurative language in non-native speakers: Erp evidence. Neurosci. Lett..

[109] Heil M., Rolke B., Pecchinenda A. (2004). Automatic semantic activation is no myth: Semantic context effects on the n400 in the letter-search task in the absence of response time effects. Psychol. Sci..

[110] Dennis T.A., Malone M.M., Chen C.C. (2009). Emotional face processing and emotion regulation in children: An erp study. Dev. Neuropsychol..

[111] Glenberg A.M., Kaschak M.P. (2002). Grounding language in action. Psychon. Bull. Rev..

[112] Bush G., Luu P., Posner M.I. (2000). Cognitive and emotional influences in anterior cingulate cortex. Trends Cogn. Sci..

[113] Kutas M., Federmeier K.D. (2000). Electrophysiology reveals semantic memory use in language comprehension. Trends Cogn. Sci..

[114] Guerra S., Ibáñez A., Martín M., Bobes M.A., Reyes A., Mendoza R., Bravo T., Domínguez M., Sosa M.V. (2009). N400 deficits from semantic matching of pictures in probands and first-degree relatives from multiplex schizophrenia families. Brain Cogn..

[115] Gunter T.C., Bach P. (2004). Communicating hands: Erps elicited by meaningful symbolic hand postures. Neurosci. Lett..

[116] Li W., Zinbarg R.E., Boehm S.G., Paller K.A. (2008). Neural and behavioral evidence for affective priming from unconsciously perceived emotional facial expressions and the influence of trait anxiety. J. Cogn. Neurosci..

[117] Helfinstein S.M., White L.K., Bar-Haim Y., Fox N.A. (2008). Affective primes suppress attention bias to threat in socially-anxious individuals. Behav. Res. Ther..

[118] Hughes G., Velmans M., De Fockert J. (2009). Unconscious priming of a no-go response. Psychophysiology.

[119] Yun X., Li W., Qiu J., Jou J., Wei D., Tu S., Zhang Q. (2011). Neural mechanisms of subliminal priming for traumatic episodic memory: An erp study. Neurosci. Lett..

[120] Lui M., Rosenfeld J.P. (2009). The application of subliminal priming in lie detection: Scenario for identification of members of a terrorist ring. Psychophysiology.

[121] Chalfoun P., Frasson C. (2010). Showing the positive influence of subliminal cues on learner’s performance and intuition: An erp study. Intell. Tutor. Syst..

[122] Eddy M., Schmid A., Holcomb P.J. (2006). Masked repetition priming and event-related brain potentials: A new approach for tracking the time-course of object perception. Psychophysiology.

[123] Holcomb P.J., Grainger J. (2007). Exploring the temporal dynamics of visual word recognition in the masked repetition priming paradigm using event-related potentials. Brain Res.

[124] Kanske P., Kotz S.A. (2007). Concreteness in emotional words: Erp evidence from a hemifield study. Brain Res..

[125] Aravena P., Hurtado E., Riveros R., Cardona J.F., Manes F., Ibáñez A. (2010). Applauding with closed hands: Neural signature of action-sentence compatibility effects. PLoS ONE.

[126] Wade N.J., Tatler B.W. (2009). Did javal measure eye movements during reading?. J. Eye Mov. Res..

[127] Rayner K., Pollatsek A. (1981). Eye movement control during reading: Evidence for direct control. Q. J. Exp. Psychol. A.

[128] Rayner K. (1998). Eye movements in reading and information processing: 20 years of research. Psychol. Bull..

[129] Yarbus A.L. (1967). Eye Movements and Vision.

[130] Allopenna P.D., Magnuson J.S., Tanenhaus M.K. (1998). Tracking the time course of spoken word recognition using eye movements: Evidence for continuous mapping models. J. Mem. Lang..

[131] Richardson D.C., Spivey M.J. (2000). Representation, space and hollywood squares: Looking at things that aren’t there anymore. Cognition.

[132] Tanenhaus M., Spivey-Knowlton M., Eberhard K., Sedivy J. (1995). Integration of visual and linguistic information in spoken language comprehension. Science.

[133] Balcetis E., David D. (2006). See what you want to see: Motivational influences on visual perception. J. Personal. Soc. Psychol..

[134] Caspi A., Hirschberger G., Ein-Dor T., Zivotofsky A.Z. (2006). Looking away from death: The influence of subliminal priming on eye movement decisions. J. Vis..

[135] Fromberger P., Jordan K., von Herder J., Steinkrauss H., Nemetschek R., Stolpmann G., Müller J. (2012). Initial orienting towards sexually relevant stimuli: Preliminary evidence from eye movement measures. Arch. Sex. Behav..

[136] Knoeferle P., Crocker M. (2009). Constituent order and semantic parallelism in online comprehension: Eye-tracking evidence from german. Q. J. Exp. Psychol. A.

[137] Knoeferle P., Crocker M.W. (2007). The influence of recent scene events on spoken comprehension: Evidence from eye movements. J. Mem. Lang..

[138] Knoeferle P., Crocker M.W. (2006). The coordinated interplay of scene, utterance, and world knowledge: Evidence from eye tracking. Cogn. Sci..

[139] Knoeferle P., Crocker M.W., Scheepers C., Pickering M.J. (2005). The influence of the immediate visual context on incremental thematic role-assignment: Evidence from eye-movements in depicted events. Cognition.

[140] Frazier L., Munn A., Clifton C. (2000). Processing coordinate structures. J. Psycholinguist. Res..

[141] Frazier L., Taft L., Roeper T., Clifton C., Ehrlich K. (1984). Parallel structure: A source of facilitation in sentence comprehension. Mem. Cogn..

[142] Blumenfeld H.K., Marian V. (2007). Constraints on parallel activation in bilingual spoken language processing: Examining proficiency and lexical status using eye-tracking. Lang. Cogn. Process..

[143] Van der Stigchel S., Mulckhuyse M., Theeuwes J. (2009). Eye cannot see it: The interference of subliminal distractors on saccade metrics. Vis. Res..

[144] Mirman D., Yee E., Blumstein S.E., Magnuson J.S. (2011). Theories of spoken word recognition deficits in aphasia: Evidence from eye-tracking and computational modeling. Brain Lang..

[145] Nummenmaa L., Hyönä J., Calvo M.G. (2006). Eye movement assessment of selective attentional capture by emotional pictures. Emotion.

[146] Fennis B.M., Pruyn A.T.H. (2007). You are what you wear: Brand personality influences on consumer impression formation. J. Bus. Res..

[147] Fitzsimons G.M., Chartrand T.L., Fitzsimons G.J. (2008). Automatic effects of brand exposure on motivated behavior: How apple makes you “think different”. J. Consum. Res..

[148] Johar G.V., Sengupta J., Aaker J.L. (2005). Two roads to updating brand personality impressions: Trait versus evaluative inferencing. J. Mark. Res..

[149] Pratkanis A.R., Greenwald A.G. (1988). Recent perspectives on unconscious processing: Still no marketing applications. Psychol. Mark..

[150] Smeets M., Dijksterhuis G. (2014). Smelly primes—When olfactory primes do or do not work. Front. Psychol..

[151] Packard V. (1957). The Hidden Persuaders.

[152] Vicary J.M. (1950). How psychiatric methods can be applied to market research. Printer’s Ink.

[153] Verwijmeren T., Karremans J.C., Bernritter S.F., Stroebe W., Wigboldus D.H.J. (2013). Warning: You are being primed! The effect of a warning on the impact of subliminal ads. J. Exp. Soc. Psychpol..

[154] Vicary J.M. (1955). The circular test of bias in personal interview surveys. Public Opin. Q..

[155] Key B.W. (1973). Subliminal Seduction.

[156] Key B.W. (1976). Media Sexploitation.

[157] Thorne S.B., Himelstein P. (1984). The role of suggestion in the perception of satanic messages on rock-and-roll recordings. J. Psychol..

[158] Strahan E.J., Spencer S.J., Zanna M.P. (2005). Subliminal priming and persuasion: How motivation affects the activation of goals and the persuasiveness of messages. Applying Social Cognition to Consumer-Focused Strategy.

[159] Aarts H., Dijksterhuis A., DeVries P. (2001). On the psychology of drinking: Being thirsty and perceptually ready. Br. J. Psychol..

[160] Strahan E.J., Spencer S.J., Zanna M.P. (2002). Subliminal priming and persuasion: Striking while the iron is hot. J. Exp. Soc. Psychol..

[161] Dijksterhuis A., Aarts H., Smith P.K., Hassin R., Uleman J., Bargh J.A. (2005). The Power of the Subliminal: Subliminal Perception and Possible Application.

[162] Pratkanis A.R., Aronson E. (2001). Age of Propaganda: The Everyday Use and Abuse of Persuasion.

[163] Greenwald A.G., Spangenberg E.R., Pratkanis A.R., Eskenazy J. (1991). Double-blind tests of subliminal self-help audiotapes. Psychol. Sci..

[164] Audley B.C., Mellett J.L., Williams P.M. Self-improvement using subliminal audiotapes: Consumer benewt or consumer fraud?. Presented at the Meeting of the Western Psychological Association.

[165] Merikle P.M., Skanes H.F. (1992). Subliminal self-help audiotapes: Search for placebo effects. J. Appl. Psychol..

[166] Brannon L.A., Brock T.C., Shavitt S., Brock T.C. (1994). The Subliminal Persuasion Controversy: Reality, Enduring Fable, and Polonious’ Weasel. Persuasion: Psychological Insights and Perspectives.

[167] Baron R.M., Kenny D.A. (1986). The moderator-mediator variable distinction in social psychological research: Conceptual, strategic, and statistical considerations. J. Personal. Soc. Psychol..

[168] Conway M., Ross M. (1984). Getting what you want by revising what you had. J. Personal. Soc. Psychol..

[169] Ross M. (1989). Relation of implicit theories to the construction of personal histories. Psychol. Rev..

[170] Ross M., Olson J.M. (1981). An expectancy attribution model of the effects of placebos. Psychol. Rev..

[171] Mikulincer M., Shaver P.R. (2015). The psychological effects of the contextual activation of security-enhancing mental representations in adulthood. Curr. Opin. Psychol..

[172] McGuire A., Gillath O., Jackson Y., Ingram R. (2018). Attachment Security Priming as a Potential Intervention for Depressive Symptoms. J. Soc. Clin. Psychol..

[173] Doche-Budzynski L., Budzynski T.H. (1989). Subliminal self-esteem enhancement in adult type a males. Education.

[174] Meerman E.E., Verkuil B., Brosschot J.F. (2011). Decreasing pain tolerance outside of awareness. J. Psycholo. Res..

[175] Shanks D.R., Newell B.R., Lee E.H., Balakrishnan D., Ekelund L., Cenac Z., Kavvadia F., Moore C. (2013). Priming intelligent behavior: An elusive phenomenon. PLoS ONE.

[176] Gillis R. (2010). Ferrari finds smoke without fire. The Wall Street Journal.

[177] Gillis R., Clegg J. (2010). Ferrari scraps barcode logo. The Wall Street Journal.

[178] Weinberger J., Westen D. (2008). Rats, we should have used clinton: Subliminal priming in political campaigns. Political Psychol..

[179] Pratkanis A.R. (1992). The cargo-cult science of subliminal persuasion. Skept. Inq..

[180] Tsai M.-t., Wen-Ko L., Liu M.-L. (2007). The effects of subliminal advertising on consumer attitudes and buying intentions. Int. J. Manag..

[181] Madzharov A.V., Block L.G., Morrin M. (2015). The cool scent of power: Effects of ambient scent on consumer preferences and choice behavior. J. Mark..

